# Agreement between HRCT Imaging and Intraoperative Measurements in Predicting Stapedotomy Prosthesis Length in Otosclerosis Patients

**DOI:** 10.22038/ijorl.2025.81759.3749

**Published:** 2025

**Authors:** Mohamad Reza Afzalzadeh, Farzaneh Khoroushi, Abolfazl Zanjani Tabasi, Yazdan Gholami Chenaran, Mohsen Rajati, Hassan Mehrad-Majd

**Affiliations:** 1 *Sinus and Surgical Endoscopic Research Center, Faculty of Medicine, Mashhad University of Medical Sciences, Iran. *; 2 *Department of Radiology, Faculty of Medicine, Mashhad University of Medical Sciences, Mashhad, Iran.*; 3 *Student Research Committee, Faculty of Medicine, Mashhad University of Medical Sciences, Mashhad, Iran*; 4 *Clinical Research Development Unit, Ghaem Hospital, Mashhad University of Medical Sciences, Mashhad, Iran.*

**Keywords:** Otosclerosis, stapedotomy, incus bone, CT scan

## Abstract

**Introduction:**

This study aimed to evaluate the accuracy of preoperative high-resolution computed tomography (HRCT) imaging in measuring the distance from the long process of the incus to the footplate and its potential for predicting the optimal prosthesis length required for stapedotomy in patients with otosclerosis.

**Materials and Methods:**

This cross-sectional study included fifty patients scheduled for primary stapedotomy. A radiologist obtained and reconstructed preoperative HRCT scans of the temporal bone to measure the distance from the long process of the incus to the oval window in both axial and coronal views. These HRCT-derived measurements were then compared with intraoperative measurements performed by an otolaryngologist. The agreement between the two methods was assessed using correlation and Bland-Altman analysis.

**Results:**

The mean distances measured by HRCT and intraoperatively were 4.15mm and 4.27mm, respectively. A strong and statistically significant correlation (r=0.928, P<0.001) was observed between the two approaches, indicating a robust association. The Bland-Altman analysis revealed a mean bias of 0.11±0.07mm, with limits of agreement (LoAs) ranging from -0.02 to 0.26 mm, and no points exceeding the 95% LoAs. The maximum potential error between the two measurement methods was 0.28mm, suggesting that HRCT imaging can reliably predict prosthesis length. In a stratified analysis based on the surgical distance (≤4 mm [N=11], 4.25mm [N=25], ≥4.5mm [N=13]), good agreement was maintained in the Bland-Altman analysis.

**Conclusion:**

Preoperative HRCT imaging may be a valuable tool for accurately predicting the required prosthesis length prior to stapedotomy in otosclerosis patients.

## Introduction

Otosclerosis is a significant cause of sensorineural hearing impairment, disproportionately affecting individuals of Caucasian descent. The condition typically begins with conductive hearing loss, which may progress to mixed hearing loss and, eventually, sensorineural hearing loss (1). 

Otosclerosis primarily impacts the otic capsule, where excessive osteoblastic activity leads to the deposition of spongy bone tissue, while osteoclastic activity destroys healthy bone tissue. This dual process ultimately leads to the fixation of the stapes, resulting in conductive hearing impairment. The most commonly affected structures include the oval window, anterior stapes, crura, footplate, and annular ligament. Conductive hearing loss occurs when the stapes, which normally transmit sound vibrations to the inner ear through the oval window, become fixed in place (2). This restriction in movement prevents the transfer of sound energy to the inner ear, leading to impaired sound conduction.

Hearing loss is a defining characteristic of otosclerosis, often presenting as a gradual decline in bilateral hearing acuity. In many instances, this deterioration is accompanied by tinnitus, a condition characterized by ringing or other sounds in the ear. Physical examination findings may be limited. Diagnosing otosclerosis typically involves a comprehensive evaluation, combining audiometry with advanced imaging techniques, including high-resolution computed tomography (HRCT) of the temporal bone. This approach allows clinicians to accurately identify the condition while ruling out other potential causes of hearing loss. (3-8). 

Patients with otosclerosis can be treated through both medical and surgical interventions. Stapedotomy, or stapedotomy with a prosthesis, is the preferred treatment option; however, complications often arise due to inaccurate prosthesis length, which is a leading cause of stapedotomy revisions.

Recent studies have shown that revision surgeries yield inferior outcomes, with success rates ranging from 16% to 80%. Each revision surgery results in a 10% reduction in the potential improvement in hearing. 

To improve the effectiveness of otosclerosis surgery, it is crucial to adhere to established surgical principles, which include ensuring appropriate indications for stapedotomy, conducting thorough preoperative evaluations using imaging techniques, and following the prescribed surgical protocols for otosclerosis procedures. Accurately measuring the prosthesis length is vital to the success of stapedotomy and revision surgeries. 

The correct prosthesis length is determined by the distance between the long process of the incus and the footplate, which serves as the standard for calculating the optimal prosthesis length. (9,10). 

Recent advances in high-resolution computed tomography (HRCT) have enabled the detection of features in the submillimeter range, significantly improving the accuracy of measurements between the long process of the incus and the footplate (11). Although there is ongoing debate regarding the necessity of performing HRCT imaging prior to stapedotomy, the current study aimed to evaluate the agreement between preoperative HRCT imaging and intraoperative measurements of the incus' long process and footplate in predicting the optimal prosthesis length for stapedotomy in otosclerosis patients.

## Materials and Methods

This study recruited 50 individuals diagnosed with otosclerosis who were referred to the Otorhinolaryngology Clinics of two major hospitals (Ghaem and Imam Reza) in Mashhad, Iran, due to complaints of hearing loss. The diagnosis of otosclerosis was confirmed after a thorough assessment, physical examination, and hearing tests. 

The inclusion criteria were as follows: a visit to the ear, nose, and throat clinic with a primary complaint of hearing loss, a definitive diagnosis of otosclerosis based on a comprehensive physical examination and hearing tests, a negative Rinne test at 512 Hz, and consent to undergo surgery. 

Exclusion criteria included patients with a history of temporal bone injuries, chronic middle ear infections, middle and inner ear anomalies, tympanosclerosis, prior surgery or disease affecting the ear, or unwillingness to continue the study. The study adhered to ethical guidelines, and all participants provided informed consent before participating. The research protocol was reviewed and approved by the ethics committee of Mashhad University of Medical Sciences, with the ethical code IR. MUMS. MEDICAL. REC.1401.579.

### Preoperative Measurements

Demographic data, including age, gender, and clinical factors such as the extent of hearing loss, were collected using a standardized checklist. 

Temporal bone CT scans were performed on patients using a Neu Viz 16 CT scanner (Neusoft Medical Systems, China) with advanced Tetrahedron Beam CT (TBCT) technology. 

**Fig 1:**
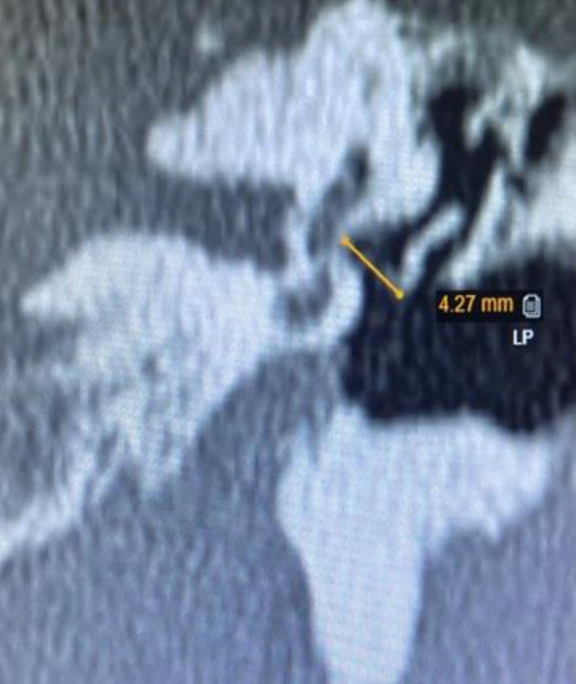
HRCT Imaging measuring a long process of incus to oval window

The CT imaging was conducted in both axial and coronal views in a Long process of incus to oval window, with a slice thickness of 1.25 mm and a collimation of 10 mm. The scan interval was set at 1.25 mm (Figure 1). 

A radiologist employed the specialized Radiant DiCom viewer software to measure the distance between the long process of the incus and the stapes footplate.

### Operation

The surgical procedure involved injecting a mixture of lidocaine and epinephrine along the perimeter of the skin in the ear canal. 

The tympanomeatal flap was elevated to gain access to the middle ear by raising the annulus. A surgical cut was made to excise the posterior superior bone of the canal, improving visibility during the procedure. The skeletal linkage was then checked for stability. 

The distance between the incus’s lateral process and the footplate’s geometric center was measured using a conductor tool (Charchon Measuring Rod) manufactured by MicroFrance. A cavity was formed in the center of the footplate, and a microdrill was used to remove the posterior cross of the stapes. 

A 0.6 mm-diameter Teflon-coated prosthetic piston was carefully inserted between the hole in the footplate and the long process of the incus. The stapes superstructure was fractured and removed by downward movement. A blood or tissue graft was placed around the prosthesis to enhance its durability. The surgeon measured the exact distance between the extended incus and the footplate using a precise and calibrated instrument, known as the operator. This measurement is not influenced by the individual performing the procedure (12).

### Statistical analysis

Statistical analysis was performed using SPSS version 22 (IBM Corporation, New York, USA). Descriptive statistics presented quantitative data as means with standard deviations, while categorical data were reported as frequencies and percentages. Pearson’s correlation coefficient was employed to examine the correlation between preoperative HRCT imaging and intraoperative measurements. Using a graphical method, a Bland-Altman analysis was conducted to quantify the differences between the preoperative HRCT imaging and intraoperative measurements. This analysis allowed for the assessment of agreement between the two methods by calculating the mean bias and its limits of agreement (LoAs) with a 95% confidence interval (CI). Statistical significance was set at P < 0.05. 

## Results

This study included a cohort of 50 individuals who underwent primary stapedotomy, with a specific focus on the distance between the long process of the incus and the stapes footplate. The baseline characteristics of the participants are presented in Table 1. The mean age of the participants was 38.52 years, with an age range from 18 to 57 years. Most participants were female, accounting for over 60% of the cohort. 

**Table 1:** Baseline characteristics of the study population.

**variable**	**Mean ± Sd / number (%)**	**minimum**	**maximum**	**P-value**
Gender (number (%))				
Males Females	20 (40) 30 (60)			
Age (years) Males Females	38.52 ± 8.29 35.25 ± 9.73 40.70 ± 6.67	18 18 26	57 50 57	0.037

**Fig 2:**
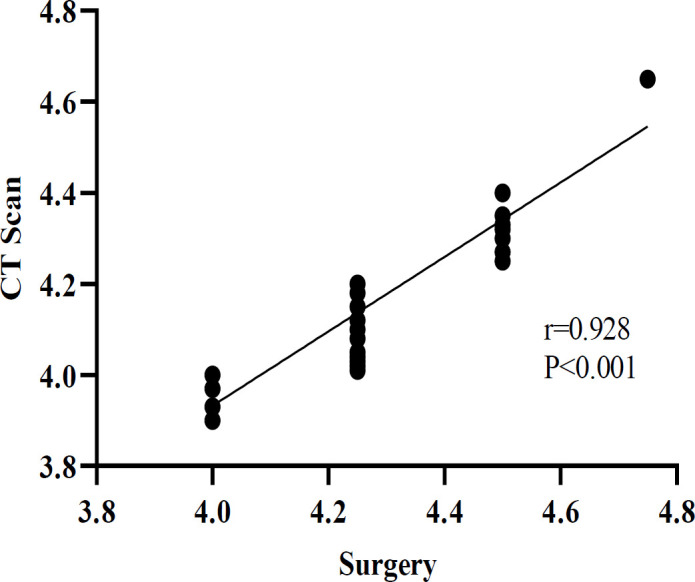
Scatter plot of correlation between CT scan and surgical measurements of long process-footplate distance

Two separate approaches were utilized to measure the distance between the long process of the incus bone and the stapes footplate: preoperative HRCT imaging and intraoperative measurements. The average distance measured by these two methods was 4.15 ± 0.17 mm and 4.27 ± 0.19 mm, respectively. The Pearson correlation coefficient revealed a substantial and statistically significant correlation (r = 0.928, P < 0.001) between the values obtained from these two methods (Figure 2).

**Fig 3:**
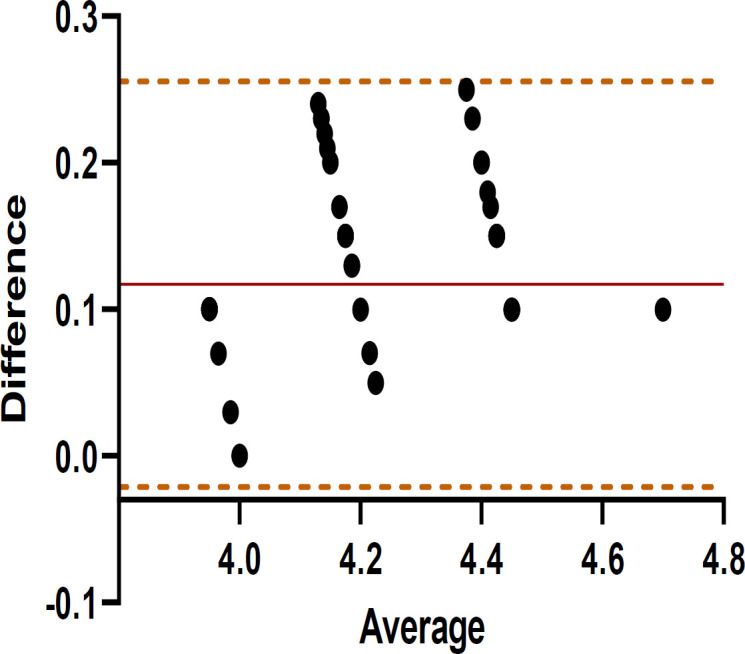
Bland-Altman plot for agreement analysis between preoperative HRCT Imaging and Intraoperative Measurements. Bias is shown as solid red line with 95% confidence intervals as dashed lines.

Bland-Altman analysis assessed the agreement and discrepancy between the two measurement approaches (Figure 3). The mean distance measured by both methods was plotted against the variation between the two methods. The results revealed a mean bias of 0.12 ± 0.07 mm, with limits of agreement (LoA) ranging from -0.02 to 0.26 mm, indicating a maximum possible discrepancy of 0.28 mm between the two measuring techniques.

The patients were divided into subgroups based on their surgical distance score to investigate further the agreement between the HRCT scan method and surgery (Table 2). 

**Table 2:** Bland-Altman analysis for groups based on the scores obtained during the operation

**Agreement between CT scan and surgery**	**Bias ± SD**	**95 % CI**	
		**lower**	**upper**
In total (N=50)	0.11 ± 0.07	-0.02	0.26
Surgical distance score ≤ 4 mm (N=11)	0.05 ± 0.04	-0.03	0.15
Surgical distance score = 4.25 mm (N=25)	0.12 ± 0.07	-0.02	0.26
Surgical distance score ≥ 4.5 mm (N=13)	0.16 ± 0.05	0.07	0.26

**Fig 4:**
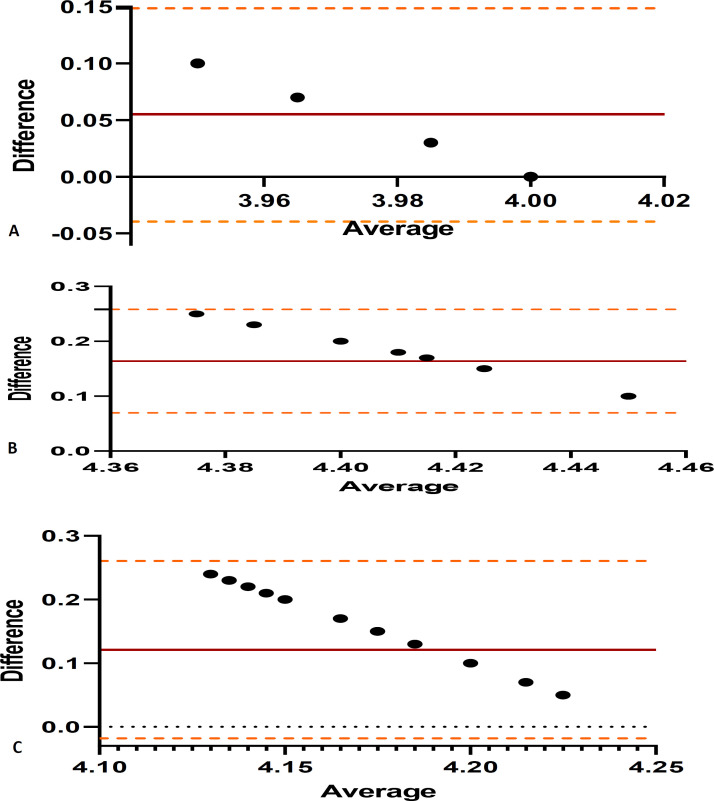
Bland-Altman plot for agreement analysis between preoperative HRCT Imaging and Intraoperative Measurements in subgroups based on the surgery score. A) Patients with a score ≤ 4 mm, B) patients with a score of 4.25 mm, and C) patients with a score ≥ 4.5 mm. Bias is shown as solid red line with 95% confidence intervals as dashed lines.

Bland-Altman analysis demonstrated varying bias levels and agreement between the methods, depending on the surgical distance score. Individuals with a surgical distance score ≤ 4 mm exhibited a minimal error tolerance between the measurement methods, with a bias value of 0.05 ± 0.04 mm and limits of agreement ranging from -0.03 to 0.18 mm (Figure 4A). 

Individuals with a surgical distance score of 4.25 mm had a bias value of 0.12 ± 0.07 mm, with limits of agreement from -0.02 to 0.26 mm, representing a discrepancy of 0.28 mm (Figure 4B). Finally, individuals with a score ≥ 4.5 mm had a bias value of 0.16 mm, with limits of agreement from -0.018 to 0.26 mm, larger than the values observed in the previous two groups (Figure 4C). 

## Discussion

The success of stapedotomy surgery is often compromised by various factors, including prosthesis displacement, incorrect prosthesis length, erosion or necrosis of the incus long process, peri-lymphatic fistula formation, and fibrous adhesion or fixation of the prosthesis (9, 13-16). Accurate measurement of the prosthesis length is critical for ensuring optimal surgical outcomes (17). 

A prosthesis that is too long can lead to excessive stimulation of the labyrinth, resulting in vertigo or instability (18-20), while a prosthesis that is too short may cause sound distortion due to inadequate conduction of the acoustic impulse to the inner ear (21-24).

The present study aimed to examine the correlation between preoperative measurement of the distance between the long process of the incus and the stapes footplate using CT scan and corresponding intraoperative measurements. The results demonstrated a strong correlation between the two methods, with a Pearson correlation coefficient of r = 0.928. Bland-Altman analysis revealed a mean bias of 0.12 mm, with limits of agreement ranging from -0.02 mm to 0.26 mm, indicating a relatively low level of disagreement between the two measuring techniques. This quantitative assessment, provided by the Bland-Altman analysis, suggests that the CT scan method can be a reliable alternative to surgical measurements. 

Subgroup analysis revealed that the agreement between the two methods was highest in patients with a distance smaller than 4 mm. This finding indicates that CT scans can be reliable for measuring this distance in patients with smaller values. In contrast, patients with larger distances (>4 mm) exhibited higher levels of discrepancy between the two methods. This increased discrepancy may be attributed to section thickness and digital image processing, which can influence visual perception on the computer screen. In the CT scan assessment of the distance between the long process of the incus and the stapes footplate, the measurement discrepancy is expected not to exceed 0.28 mm compared to the surgical measurement. From a clinical perspective, this level of disparity can be considered negligible and does not affect the feasibility of using the CT scan technique as a reliable alternative. Our findings are consistent with those reported by Gosselin et al., who identified a strong positive correlation between CT scans and surgical measurements, suggesting that CT scans can effectively measure distances before stapedotomy surgery. However, in contrast to our subgroup analysis, where patients with a distance smaller than 4 mm exhibited the highest level of agreement between surgical and CT scan measurements, Gosselin et al. found that the greatest level of agreement was observed in measurements greater than 4 mm. 

This discrepancy may be attributed to differences in the thickness of HRCT sections and the settings of the digital display on the computer screen. Similarly, Prasad et al. conducted a comparable study and reported a strong correlation and agreement between the distances calculated on CT scans and those observed during surgery (25). 

Despite existing research on the correlation between CT scans and surgical procedures for determining the optimal length of prosthesis used in stapedotomy, further investigation is necessary to increase certainty in using this approach before surgery. Future studies should aim to investigate the relationship between CT scan and surgical measurements in a larger sample size and consider additional factors that may influence the length of the prosthesis, such as the shape and size of the incus long process (26), alterations in the angle of the incudo-stapedial joint, and the thickness of the incus long process.

## Conclusion

In conclusion, the present study highlights the potential of CT scans as a reliable and accurate method for estimating prosthesis length prior to stapedotomy in patients with otosclerosis. The results suggest that CT scans provide precise measurements of prosthesis size with minimal margin of error. While these findings offer valuable insights, further research is needed to validate and expand upon them, ultimately improving the use of CT scans as a predictive tool in otosclerosis surgery. 

Future studies should focus on exploring the correlation between CT scan measurements and intraoperative findings, as well as examining potential limitations and confounding variables that may affect the accuracy of CT scans in predicting prosthesis length.
